# Supplementary material for: The Climate and Nutritional Impact of Beef in Different Dietary Patterns in Denmark
Source: Foods. 2020 Aug 25;9(9):1176. doi: 10.3390/foods9091176 (PMC7555984; doi:10.3390/foods9091176)
Supplement: Supplementary file 1 [file foods-09-01176-s001.pdf]

Table S1. Estimated share of origin for food consumed in Denmark based on country/region of import.

**Type of Food**	**Danish %**	**Imported foods distributed on country of import, % 1)**													**Reference, Danish %**
		Sweden and Norway	Germany	The Netherlands and [illegible]	UK	Italy/Southern Europe	New Zealand	China	Greenland/Island	Canada/USA	South America	Poland	Malaysia og Asia	Russia/ Baltics	
Milk and dairy	84-90	13	50	12	13	8	2	0	0	0	0	3	0	0	2+3)
Cheese	81	13	50	12	13	8	2	0	0	0	0	3	0	0	2)
Flour (and grains)	50-87	21	29	8	0	9	0	0	0	0	0	13	0	21	4)
Bread	62-84	17	40	18	6	16	0	0	0	0	0	4	0	0	4)
Vegetables	45-90	5	33	31	0	27	0	1	0	0	0	3	0	0	5)
Fruits	0-30	4	31	25	3	30	0	0	0	2	0	4	0	0	5)
Pork	91	4	67	8	0	9	0	1	0	0	0	11	0	0	3)
Fish and sea food	58	41	7	0	4	0	0	1	29	14	0	3	0	0	3)
Beef	60	0	31	33	13	11	1	0	0	0	0	9	0	2	1)
Poultry	63	27	45	13	3	5	0	0	0	0	0	5	0	2	3)
Egg	62	23	22	34	1	0	0	0	0	4	1	7	0	7	2)
Oils and fats	0-49	14	43	6	0	7	0	0	0	0	0	0	30	0	6)
Sugar	76	20	32	21	4	20	0	0	0	0	0	5	0	0	3)
Coffee and spices	0	23	30	8	8	6	0	0	0	0	19	3	4	0	

1) Danish Agricultural and Food Council 2017b [31]. Country of import is not necessarily always the country of origin, however this was assumed here.
2) Danish Agricultural and Food Council. 2009 [32]. Data from 2008. (Butter: 49% Danish)
3) FAOStat data from 2013-14 [33].
4) FAOStat data [79]. Data for wheat and wheat products: 87% Danish, but the share is lower for Danish grain for bread – 50-80% assumed.
5) Vegetable Market Seasonal Guide [34]: Carrots 87%, potatoes 90%, onions 80%, tomatoes 46%, cucumbers 50%, peas 75%, lettuce 50%, apple and pears 19%, strawberry 30%, oranges and bananas 0%.
6) Rapeseed oil 47%, butter 49%, olive oil and other 0%.

Table S2. Typical routes of transport for imported feed and related greenhouse gas (GHG) emission per kg food from transport to Denmark.

**Import Country**	**Distance** [1)]	**Means of Transport**	**GHG Emission, CO_2eq g/kg Food** [2)]
Sweden	*300 km*	*Truck (Linköking-Göteborg)*	55
	278 km	*Ship (Göteborg-Århus)*	
Germany	*650 km*	*Truck (Kassel-Århus)*	78
The Netherlands	*100 km*	*Truck (to Amsterdam)*	65
	1015 km	*Ship (Amsterdam-Århus)*	
France	*450 km*	*Truck (to Le Havre)*	117
	1430 km	*Ship (Le Havre-Århus)*	
Spain	*450 km*	*Truck (to Gijón)*	154
	2335 km	*Ship (Gijón-Århus)*	
Italy	*250 km*	*Truck (to Naples)*	256
	5310 km	*Ship (Naples-Århus)*	
Poland	*340 km*	*Truck (to Gdansk)*	76
	702 km	*Ship (Gdansk-Århus)*	
Russia	*500 km*	*Truck*	160
	1000 km	*Train (to Riga)*	
	1085 km	*Ship (Riga-Århus)*	
USA	*600 km*	*Truck*	251
	1300 km	*Train (to Norfolk)*	
	6569 km	*Ship (Norfolk-Rotterdam)*	
	1082 km	*Ship (Rotterdam-Århus)*	
Argentine	*700 km*	*Truck (to Buenos Aires)*	257
	11744 km	*Ship (Buenos Aires-Rotterdam)*	
	1082 km	*Ship (Rotterdam-Århus)*	
Brazil	*1200 km*	*Truck (to Santos)*	288
	10056 km	*Ship (Santos-Rotterdam)*	
	1082 km	*Ship (Rotterdam-Århus)*	
Indonesia	*900 km*	*Truck (to Balikpapan)*	337
	17229 km	*Ship (Balikpapan-Rotterdam)*	
	1082 km	*Ship (Rotterdam-Århus)*	
Malaysia	*400 km*	*Truck (til Miri)*	323
	16536 km	*Ship (Miri-Rotterdam)*	
	880 km	*Truck (Rotterdam-Århus via Fyn)*[)]	
Australia	*500 km*	*Truck to Perth*	303
	17733 km	*Ship (Perth-Rotterdam)*	
	1082 km	*Ship (Rotterdam-Århus)*	

1) Calculated by means of http://www.sea-distances.org and https://www.google.dk/maps from import country to harbor of Aarhus, Denmark

2) GHG from transport regarding import : Ship transport: national 45 g CO_2/tkm, European: 41 g CO_2/tkm, overseas: 11 g CO_2/tkm (Agri-footprint, 2015 [35]); by train in Europe: 52 g CO_2/tkm and train in the rest of the world: 47 g CO_2/tkm (Ecoinvent3, 2013 [36]); transport by truck from 100-370 g CO_2/tkm depending on truck size (Agri-footprint, 2015 [35]).

Table S3. Estimate for greenhouse gas (GHG) emission related to storage in supermarket and home.

Type of storage in Supermarket and Home	GHG Emission, g CO_2eq/kg Food	Type of Storage Used per Food Group
100% dry [1)]	**60**	Coffee, sugar, rice, pasta
80% dry and 20% cold	68	Fruit, potatoes, soft drink, alcohol,
100% cold	**100**	Dairy products, eggs,
80% dry and 20% frozen	143	Breed, bakery
60% dry, 20% cold, 20% frozen	151	Vegetables, legumes, berries
80% cold and 20% frozen	175	Meat, fish
100% frozen	**475**	Dessert (mainly ice cream)

1) Mainly general energy use in supermarket.

Table S4. Energy use for cooking, weight loss during cooking and greenhouse gas (GHG) emission from food preparation for different types of beef products.

Type	Cooking	Recipe	Time	Cooking-Loss [1], %	GHG Emission [2], CO_2eq. g/kg	Reference
**Minced**	Fried on pan [3]	Minced meat	12 min [4]	28-29	409	1)
	Cooked in stew	700 g of beef [8]	25 min	(20)	370	5)
	Baked in oven	1 kg meat	45-50 min	(20)	534	estimate
		Average applied			410	6)
**Roast**	In oven	1 kg at150 degree C	60-70 min.	15-20	672	1)
		1 kg at 180 degree C	60 min	20-25	19	1)
	Roast in pan	1 kg	45 min	20-25	341	1)
		Average applied			490	7)
**Strips**	Cooked in stew	700 g of beef [8]	25 min	(20)	370	5)
**Steaks**	Pan [3]	1½-2 cm thickness	8 min.	15-20	312	1)

1) Institute of Technology (2017 personal communication)[38].
2) 1 kWh electricity = 3.6 MJ out, 172 g CO_2/MJ electricity (Danish average mix)(Ecoinvent3, 2013 [36]) resulting in 619 g CO_2/kWh.
3) 500 g at a time.
4) 10 min high temperature – 2 min low temperature, in total 334 W/0.5 kg beef.
5) Thy Mors Energy [39].
6) Assumption: 35% in pan, 50% cooked in stew, 15% in oven.
7) Assumption: 50% in oven and 50% in pan.
8) In total 1.13 kg dish.

Table S5. Energy use for cooking and greenhouse gas (GHG) emission from cooking for different types of food items [1)]

Type of Cooking	Example	Energy Use	GHG Emission g CO_2eq./kg Raw Food
**Boiling**			
Much water (food: water 1:3)	250 g pasta and 750 g water	1.06 kWh/kg food	656
Medium water (food: water 3:5)	300 g rice and 500 g water	0.71 kWh/kg food	438
Little water (food: water 5:1)	1 kg vegetables and 200 g water	0.32 kWh/kg food	197
**Heating water**			
Coffee maker	1 kg	0.155 kWh/kg	96
Electric kettle		0.119 kWh/kg	74
**Oven**			
1 hour in oven	1 kg food	1 kWh	619
Baking a cake	25 minutes	0.39 kWh/kg food	242

1) Energy use based on Measurement from Thy Mors Energy (2014) [39].

Table S6. Avoidable food waste in the chain - from different references and used in this study, % weight loss.

	Retail [2)]	Household					Used in This Study			
**References** [1)]	PEF, 2017	Thorsen et al., 2012	Wrap, 2008	Wrap, 2014	PEF, 2017	Petersen et al., 2015	**Processing**	**Retail**	**House hold**	**Total**
Milk and dairy products	0.5	6	3	7	7	(1)	2	3	3	8
Cheese and cheese products	0.5	6	3	7	7	5	2	3	3	8
Breakfast cereals	1	2	15	7	2	3	2	2	2	6
Bread	2	6	31	22	25	14	2	6	19	27
Rice, dry	1	2	15	7	2	9	2	2	2	6
Pasta, dry	1	2	15	7	2	9	2	2	2	6
Vegetables	10	6	19	21	19	14	2	6	19	27
Potatoes	10	6	19	21	19	7	2	6	19	27
Fruit	10	6	26	14	19	6	2	6	19	27
Nuts/almonds	1	2	15	14	2	6	2	2	2	6
Juice	1	2	18	7	5	1	2	2	5	9
Meat	4	6	13	11	11	10	2	2	11	15
Fish and fish products	4	6	13	11	11	10	2	2	11	15
Egg	1	2	18	7	2	6	2	2	2	6
Dressing	1	2	18	13	4	-	2	2	4	8
Fat	1	2	3	4	4	6	2	2	4	8
Sucker	5	2	17	7	2	8	2	2	2	6
Honey and marmalade	5	2	17	7	2	8	2	2	2	6
Ice, sweets and chocolate	5	2	3	7	2	8	2	2	2	6
Cakes	5	2	17	11	25	8	2	6	19	27
Drinks	1	2	3	7	5	-	2	2	5	9

1) PEF, 2017: Guidance for the development of Product Environmental Footprint Category Rules) [67], Thorsen et al., 2012 [68]
WRAP 2008 [69]
WRAP 2014 [70]
Petersen et al., (2015) [71]—for fruits and vegetables numbers from Petersen et al., 2015 was raised with 15 % for including the proportion of waste that was ending as compost, food waste for vegetable upgraded from 12 to 14%, and food waste for fruit upgraded from 5 to 6%.

2) Include supermarket and storage at grocery.

Table S7. Unavoidable food waste in household—cleaning waste (non-edible) and weight loss at cooking.

Reference	Foodcomp (2014) [74]		McCance and Widdowson (2015) [75]	Used in This Study	
**Weight loss (%)**<br>**Food group**	**Cleaning**	**Cooking**	**Cleaning**	**Cleaning**	**Cooking**
Milk and milk product	0	0		0	0
Cheese and cheese product	0	0		2	0
**Bread and cereals**					
Cereals breakfast	0	0		0	0
Bread	0	0		0	0
**Rice and pasta**	0	0		0	0
**Vegetables**					
Carrots [1)]	15	5	13-29	15	5
Pea raw/frozen	60/0	5	63/0	60/0	5
Cabbage(white)	20	5	9-10	15	5
Beet root, fresh	20	5	20	20	5
Onion	10	5	10	10	5
Lettuce	5-10	0	18	15	0
Tomato	0	5	1	0	5
Cucumber	5	0	8	8	0
Bell pepper	10	5	14	10	5
Leek	15	5	43	20	5
**Potatoes** [1)]	15	5	5-15	15	5
**Fruit (not including juice)**					
Apple/pear	15	0	13	15	0
Orange	29	0	29	29	0
Banana	40	0	47	40	0
Strawberries	3	5	6	5	5
Nuts/almonds	5	0	-	5	0
**Juice**	0	0		0	0
**Meat**					
Pork	0	25-30		0	20
Beef, minced [2+5)]	0	15-29		0	20
Beef, roasted [3+5)]	2-15	15-25		2-15	20
Beef, steak [5)]	1	15-20		1	20
Beef, dice/strips [4)]	1	15-20		1	20
Beef, giblets [6)]	7	20		7	20
Poultry meat, skin/meat	30/42	20/15		30/42	20/15
**Fish and fish products**					
Herring, fillets/untreated	0/35	0/20		0/35	0/20
Shrimps, peeled	0	17		0	17
Cod, fillets	0	20		0	20
Flat fish, flounder untreated	67	20		67	20
Eggs	11	0		11	0
Dressing	0	0		0	0
Fats (on bread)	0	0		0	0
Fats (other)	0	0		0	0
Sugar	0	0		0	0
Honey and jam	0	0		0	0
Ice-cream, sweets, chocolate	0	0		0	0
Cake	0	0		0	0
Fast-food	0	0		0	0
**Beverages**					
Coffee	0	0		0	0
The	0	0		0	0
Alcohol	0	0		0	0
Soft drinks	0	0		0	0

1) For new carrots and potatoes freshly harvested, and for old carrots and potatoes from store, respectively.
2) Lean minced meat.
3) Rib-eye (lowest number) and brisket of beef (highest number) respectively.
5) Lean – medium fat content.

6) Data from Danish Technological Institute (Bejerholm, DMRI, 2017, pers. Com.) [38].
7) Data for calf's liver.

Table S8. Carbon footprint (CF) and arable land use (LU) per kg food item appearing in the supermarket and per kg food on the plate, respectively—taking into account food losses in the chain

	CF, g CO_2eq. and LU, m^2, per kg Food Appearing in Supermarket						CF, g CO_2eq. and LU m^2, per kg Food at the plate				Import, %	Cooked, %	Reference
	CF					LU	CF			LU			
	Farming processing packing [1]	Trans-port [2]	Storage [3]	Losses [4]	**Total CF**	**m^2** [5]	Cooking [6]	**Losses** [7]	**Total CF**	**m^2** [8]			
**1.Mik product**													
Whole milk	1090	79	100	66	1335	1.16	0	41	1376	1.19	10	0	9)
Semi skimmed milk	920	79	100	57	1156	0.98	0	36	1192	1.01	10	0	9)
Skimmed milk	860	79	100	54	1093	0.92	0	34	1127	0.94	10	0	9)
Milk yogurt and other	1200	79	100	72	1451	1.27	0	45	1496	1.31	10	0	9)
**2.Cheese** (yellow)	9501	157	100	507	10265	10.41	0	533	10799	10.96	19	0	9)
**3.Cereals and bread**													
Oat flakes	730	94	60	36	920	2.11	0	19	939	2.16	32	0	10)
Wheat bread	840	87	143	92	1161	1.34	0	272	1434	1.65	27	0	11)
Rye bread	716	87	143	81	1027	1.41	0	241	1267	1.74	27	0	11)
Rice (uncooked)	2900	141	60	128	3229	2.49	447	74	3751	2.54	100	100	12)
Pasta (uncooked)	1230	87	60	57	1433	4.57	669	29	2132	4.66	27	100	13)
**4.Vegetables**													
Carrots	142	58	100	26	326	0.,31	178	165	668	0.47	13	60	14)
Potatoes	178	54	100	28	356	0.29	384	196	941	0.45	10	100	10)
Pea and other	218	73	151	38	480	2.71	73	112	665	3.35	25	30	15)
Cabbage	220	110	68	34	432	0.21	209	222	863	0.31	55	70	16)
Beetroot	379	42	68	42	531	0.33	290	337	1158	0.53	0	90	18)
Onion	379	67	68	44	558	0.33	271	250	1078	0.47	20	95	14)
Lettuce	469	104	68	55	695	0.33	0	315	1010	0.47	50	0	16)
Tomatoes	525	108	68	60	761	0.02	99	198	1058	0.03	54	40	16)
Cucumber and other [17]	525	104	68	60	756	0.02	0	259	1015	0.03	50	0	16)
**6. Fruit**													
Orange	360	172	68	51	651	0.54	0	481	1133	0.94	100	0	19)
Banana and other exotic [21]	400	172	68	51	695	0.54	0	735	1430	1.12	100	0	20)
Apple, pear and other [23]	200	147	68	36	451	0.54	0	204	655	0.79	81	0	22)
**7. Juice**	740	172	60	40	1012	0.52	0	53	1065	0.55	100	0	24)
**8. Meat**													
Beef	13030	140	175	550	13895	14.84	557	5961	20413	21.20	44	100	25)

Pork	4400	110	175	193	4879	7.71	548	1973	7400	10.82	9	100	26)
**9. Fish**	4450	164	175	197	4986	0.00	516	1605	7107	0.00	42	100	27)
**10. Poultry**	4410	132	175	195	4912	7.60	516	1587	7035	10.05	37	100	28)
**11. Eggs**	1958	81	100	88	2228	3.64	226	326	2780	4.18	48	100	10)
**12. Fats**													
Butter	10470	186	100	443	11199	11.01	0	467	11666	11.46	51	0	9)
Vegetable oil and other	2024	127	68	91	2310	6.74	185	96	2591	7.02	53	90	29)
**13. Sugar and sweet**													
Sugar	571	67	60	29	727	0.91	125	15	867	0.92	24	50	30)
Sweet	2383	67	60	104	2614	3.60	0	53	2667	3.68	24	0	31)
**14. Beverages**													
Beer	1000	63	68	47	1177	0.52	0	62	1239	0.55	18	0	32)
Wine	2100	218	68	98	2484	1.56	0	131	2615	1.64	100	0	33)
Alcohol, e.g. whiskey	2967	130	68	131	3296	1.56	0	173	3469	1.64	50	0	34)
Lemonade (ready to drink)	159	77	68	13	317	0.18	0	17	333	0.19	20	0	35)
Soft drinks e.g. cola	300	77	68	18	464	0.18	0	24	488	0.19	20	0	36)
Water (tap)	1	0	0	0	1	0	0	0	1	0	0	0	37)
Bottled water	101	62	60	9	232	0	0	12	244	0	20	0	37)
Coffee (ready to drink)	160	16	60	10	246	0.31	101	13	360	0.33	100	100	38)
Tea (ready to drink)	46	5	60	5	116	0.08	78	6	200	0.09	100	100	39)

1) The reference number given in the column ‘Reference’ relate to the reference for carbon footprint from farming, processing and packing per 1 kg food item produced.
2) CF from transport taking into account the proportion of this food which is imported (column ‘import’ gives the proportion of each food item assumed imported.
3) CF from storage per food group is given in Supplementary Table S3.
4) Carbon footprint for farming, processing, transport and storage of the extra amount of food that need to be produced to have 1 kg food appearing in supermarket (the applied % losses in processing and retail per food item given in Supplementary Table S5).
5) The reference for LU per food item is as a starting point the same reference used for CF from farming etc—more detail about references hereof given in the text.
6) CF of cooking include also energy for cooking of the extra amount of food due to losses – both the unavoidable weight loss from cooking and avoidable loss in household, which was assumed to happen after cooking.
7) CF from extra amount of food due to losses in household—more kg needed from supermarket due to this avoidable and unavoidable loss in household.
8) LU—taking into account extra amount that need to be produced due to losses in the chain.
9) Flysjö, 2012 [40]. CF of milk yoghurt was also used as the estimate for CF of crème fraiche and cream and CF of yellow cheese was used as estimate for CF of all cheese as this type of cheese contribute around 90% of cheese intake.
10) Nielsen et al., 2003 [41].
11) Nielsen et al., 2003 [41], Werner (2014) [42].
12) Kasmaprapruet et al. (2009) [43].

13) Heusala et al., 2020 [44].
14) Halberg et al., 2006 [45].
15) Landquist, 2012 [46]. The same CF estimate was used as estimate for beans.
16) Davis et al., 2011 [47].
17) CF of cucumber was used as a CF estimate for other vegetables: bell pepper, squash, eggplant, mushroom, asparagus, olive, avocado, celery, corn.
18) CF for onion was used as an estimate for beetroot.
19) Sanjuàn et al., 2005 [48].
20) Luske, 2010 [49].
21) CF of banana was also used as an estimate for CF of other imported fruit; pineapple, kiwi, melon, nectarine, peach, grape.
22) Audsley et al., 2009 [50].
23) CF of apple and pear was used as an estimate for CF of berry.
24) Mordini et al., 2009 [51].
25) Mogensen et al. 2015 [52], Mogensen et al. 2016 [22].
26) 3.4 kg CO_2/kg carcass from Nguyen et al., 2011 [53] transformed to 4.25 kg CO_{2eq}/kg meat (1.25 as meat conversion factor from carcass to bone-free meat (Hartikainen and Pulkkinen, 2016 [37]).
27) For fish was used CF values for filet (Thrane, 2004 [54]) i.e. meat. This estimate was based on an assumed mix of fish intake; 1/3 cod, 1/3 flatfish and 1/3 herring.
28) Leinonen et al., 2012 [55].
29) As an estimate for CF of vegetable oil was used CF of Danish rapeseed oil (Mogensen et al., 2014 [56]). This CF estimate was also used as an estimate for margarine and mayonnaise.
30) Mogensen et al., 2018 [57].
31) Intake of sweet is a mixture of cake (45-56% of total amount of sweet across different diets) candy (25-38%) and ice cream (16-19%). As an general CF estimate for sweet was used CF of sweet of 2.5 kg CO_{eq}/kg from SIK (2010) [58].
32) Hanssen et al., 2007 [59].
33) Ardente et al., 2006 [60].
34) Garnett, 2007 [61].
35) Based on assumptions; ¼ lemonade (50:50 berry and sugar) and ¾ water.
36) Amienyo et al., 2013 [62], Sinclair, 2009 [63]; Röös, 2014 [64].
37) Wallman and Nielsen, 2011 [65].
38) 45 g coffee beans /l coffee, CF 3.6 kg CO_{eq}/kg beans (Hanssen et al., 2007) [59].
39) 13 g tea leaves/l tea, same CF as coffee beans assumed.
